# Preparing medical students for obstetrics and gynecology milestone level one: a description of a pilot curriculum

**DOI:** 10.3402/meo.v19.25746

**Published:** 2014-11-26

**Authors:** Helen Morgan, David Marzano, Michael Lanham, Tamara Stein, Diana Curran, Maya Hammoud

**Affiliations:** 1 Department of Obstetrics and Gynecology, University of Michigan Medical School, Ann Arbor, MI, USA; 2 Department of Medical Education, University of Michigan Medical School, Ann Arbor, MI, USA; 3 Reproductive Endocrinology and Infertility, Department of Obstetrics and Gynecology, University of Michigan Medical School, Ann Arbor, MI, USA; 4 Faculty in the Division of Anatomic Sciences, Department of Medical Education, University of Michigan Medical School, Ann Arbor, MI, USA

**Keywords:** ACGME Milestones, flipped classroom, active learning

## Abstract

**Background:**

The implementation of the Accreditation Council for Graduate Medical Education (ACGME) Milestones in the field of obstetrics and gynecology has arrived with Milestones Level One defined as the level expected of an incoming first-year resident.

**Purpose:**

We designed, implemented, and evaluated a 4-week elective for fourth-year medical school students, which utilized a multimodal approach to teaching and assessing the Milestones Level One competencies.

**Methods:**

The 78-hour curriculum utilized traditional didactic lectures, flipped classroom active learning sessions, a simulated paging curriculum, simulation training, embalmed cadaver anatomical dissections, and fresh-frozen cadaver operative procedures. We performed an assessment of student knowledge and surgical skills before and after completion of the course. Students also received feedback on their assessment and management of eight simulated paging scenarios. Students completed course content satisfaction surveys at the completion of each of the 4 weeks.

**Results:**

Students demonstrated improvement in knowledge and surgical skills at the completion of the course. Paging confidence trended toward improvement at the completion of the course. Student satisfaction was high for all of the course content, and the active learning components of the curriculum (flipped classroom, simulation, and anatomy sessions) had higher scores than the traditional didactics in all six categories of our student satisfaction survey.

**Conclusions:**

This pilot study demonstrates a practical approach for preparing fourth-year medical students for the expectations of Milestones Level One in obstetrics and gynecology. This curriculum can serve as a framework as medical schools and specific specialties work to meet the first steps of the ACGME's Next Accreditation System.

Milestones, as defined by the Accreditation Council for Graduate Medical Education (ACGME), are competency-based, developmental outcome expectations that can be demonstrated progressively by residents and fellows from the beginning of their education, through graduation, and then into unsupervised practice in their specialty. The Milestones Project is a part of the ACGME's Next Accreditation System (NAS) (1), and was implemented in obstetrics and gynecology in July 2014. Milestones specific for obstetrics and gynecology were defined and described by a joint initiative consisting of the ACGME, the American Board of Obstetrics and Gynecology (ABOG), and the American College of Obstetrics and Gynecology (ACOG); and were released in September 2013 (2). Milestones Level One are defined as the level expected of an incoming first-year resident.

At this time, there is a lack of formal curricula targeting Milestones Level One for fourth- year medical students planning to enter obstetrics and gynecology residencies. Although some programs have implemented intern preparatory courses as part of their residency programing, there is a need for a comprehensive program for educating and assessing graduating seniors (3), and for facilitating their transition to residency (4). The University of Michigan Department of Obstetrics and Gynecology designed, implemented, and evaluated a 4-week elective, which utilized a multimodal approach to teaching and assessing the Milestones One competencies. The objective of this paper is to provide a detailed description of the pilot curriculum.

## Methods

### Course design

The curriculum was organized by an education team that consisted of leadership from the Residency program, the Medical Student Clerkship, and the Department of Medical Education. Resident and medical student feedback was also obtained during monthly planning meetings. The 4-week, 78-hour curriculum utilized traditional didactic lectures, flipped classroom active learning sessions, a simulated paging curriculum, simulation training, embalmed cadaver anatomical dissections, and fresh-frozen cadaver operative procedures. The content for the learning objectives was initially mapped to the preliminary version of the Obstetrics and Gynecology Milestones as outlined by the American Professors of Gynecology and Obstetrics (APGO) and the Council on Resident Education in Obstetrics and Gynecology (CREOG) Milestones One Educational Taskforce (Table 1) (5). Five fourth-year medical students, who all matched in obstetrics and gynecology, participated in the March 2013 course. Faculty were recruited from the Departments of Obstetrics and Gynecology, Anesthesia, Pediatrics, Risk Management, and Nursing. IRB exemption was obtained by the University of Michigan Medical School Institutional Review Board.

**Table 1:** Teaching modality utilized for topics in the Advanced Clinical Skills in Obstetrics and Gynecology course, University of Michigan, 2013

Topic	Didactic lectures	Flipped classroom sessions	Simulated paging curriculum	Simulation training	Anatomic dissection	Operative procedures
Obstetrical ultrasound	×			×		
Basic laparoscopy				×		
Coding and billing	×					
Vulva disorders		×				
Communication and professionalism	×					
Leadership	×					
Anatomy of the abdominal wall					×	×
Pelvic floor anatomy					×	×
Pelvic vasculature					×	×
Perineal anatomy					×	×
Basic hysteroscopy				×		
Labor and delivery				×		
Gynecological oncology cases		×	×			
Informed consent	×					
Intern level operative procedures						×
Advanced operative procedures						×
First trimester bleeding		×				
Prolapse and incontinence		×				
Obstetrical anesthesia	×					
Disorders of menses	×					
Medical complications of pregnancy	×					
Contraception	×					
Gynecological office procedures				×		
Approach to the literature		×				
Gynecological office management cases				×		
Postpartum complications			×			
Gynecological postoperative care	×		×			
Prescription writing	×					
Wound care	×					
Obstetrical triage	×					
Operative positioning				×		

#### Didactic lectures

One-third of the curriculum was delivered in a traditional didactic format including PowerPoint presentations and case discussions. Topics delivered in this method included general topics such as diabetes and hypertension in pregnancy, gynecologic wound care, contraception, and informed consent.

#### Flipped classroom sessions

The flipped classroom curricular model was utilized for the topics of prolapse and incontinence, gynecological oncology cases, vulvar disorders, first trimester bleeding, and gynecologic office management cases. For these sessions, students were assigned content to review prior to the teaching session. This preclass assignment consisted of either reviewing a high content video, or completing a reading assignment. Classroom time was then used for the application of knowledge in the form of a case-based active learning session. We utilized the open source learning design site Learning Activity Management System (LAMS) (6) for these sessions. The LAMS program enables each student to respond individually to short answer questions, while working through clinical cases together as a group. Students replied individually to short answer prompts on the differential diagnoses, laboratory evaluation, and management of the cases. Collective responses were then projected anonymously and used for discussion.

#### Simulated paging curriculum

A paging curriculum consisting of four gynecological and four obstetrical patient scenarios was created and modeled after a similar paging curriculum used in a surgery residency preparation course (7). The scenarios consisted of common paging scenarios such as tachycardia in the postoperative unit, or postpartum urinary retention (Table 3). Eight certified nurse midwifes (CNM) were recruited to page learners at randomly selected times, 24 hours a day, 7 days a week over the 4-week period. The CNM, playing the role of a clinical bedside nurse, used standardized scripts containing relevant clinical information. Specific details were only conveyed if specifically requested by the student. The scenario ended with the completion of the call, and immediate feedback was given to the student. A paging debrief session was held the last week of the course to review the eight cases and reinforce the important clinical teaching points. A paging confidence survey was administered on the first and last day of the elective.

#### Simulation training

A selection of clinical scenarios that utilized obstetrics and gynecology simulation cases, previously used in our residency curriculum (8), served as the basis for the simulation sessions. For obstetrical simulations, SimMom^®^ and Noelle^®^ human birth simulators were utilized. Traditional box trainers were used for hysteroscopic and laparoscopic simulations. For each clinical case, a short didactic presentation was made on the clinical presentation of each scenario. Each participant was then given a brief clinical vignette, and instructed to manage the patient for the particular scenario. Items for evaluation included knowledge of clinical situation, recognition of pertinent signs of the clinical problem, management, and team communication where applicable. Upon completion of each session, learners were given direct feedback in each area. Learners were then given the opportunity to repeat the station.

#### Anatomic dissections

Basic pelvic anatomy sessions, utilizing embalmed cadavers, were designed and conducted by anatomy department faculty. These sessions covered layers of the abdominal wall, pelvic viscera and blood supply, pelvic floor muscles, and perineal anatomy. One faculty member from the Division of Anatomical Sciences taught all five sessions.

#### Operative procedures

The clinical anatomy sessions utilized fresh-frozen cadaveric pelvises and were conducted by two obstetrics and gynecology faculty. These sessions covered two basic first-year level surgical cases (tubal ligation and second-degree perineal laceration), and two advanced level cases (abdominal hysterectomy and bilateral salpingo-oophorectomy) to reinforce the clinical relevance of the pelvic anatomy sessions. The fresh-frozen dissections were performed over a 2-day period with a cadaver-to-student ratio of 1:2.5. Instruction included an introduction to the surgical instruments and sutures used for each procedure as well as a brief review of the clinical anatomy. Students then performed a tubal ligation, a second-degree perineal laceration repair, and a total abdominal hysterectomy with bilateral salpingo-oophorectomy on their cadavers. Learners were given feedback as to economy of moves, proper use of instruments, and quality of knots tied. Throughout each procedure, common complications and potential variations in anatomy and technique were described. The cost per fresh cadaver was $1,245 each, and the surgical instruments from the university operating rooms were used at no cost.

### Student assessment

#### Knowledge

A 100-question knowledge assessment for all educational objectives domains was constructed based on content drawn from current ACOG Practice Bulletins and Committee Opinions, peer-reviewed publications, and traditional textbook chapters. The multiple-choice test was administered via Qualtrics^®^ on the first and last day of the course. Excel^®^ was used to create a report of test scores, a list of all educational outcomes, and a personalized reading list of references for all missed questions. Each student had an individual feedback session after the knowledge pretest, where they were given their report with their test scores, their personalized reading lists, and recommended course of study to be completed over the 4-week period.

For the paging curriculum, a pre- and postparticipation paging confidence assessment was administered using SurveyMonkey^®^. Students rated their confidence in handling pages related to the specific gynecological and obstetrical scenarios on a six-point Likert scale (0=Not at all, to 6=I can teach someone how to do it). In addition, scoring rubrics that evaluated participants on assessment and management for each scenario were developed and the CNM who performed the paging scenario filled out the evaluation on the student.

#### Skills

An open skills assessment was performed on the first and last days of the course, using an abbreviated version of a previously validated open skills curriculum (9). Students were timed performing a two-handed knot tie without tension, one-handed knot tie without tension, two-handed surgeon's knot under tension, simple interrupted suture, and simple running suture. Laparoscopic skills assessment was performed with a pre- and posttest utilizing basic laparoscopy skill on a box trainer. Students were given a surgical kit for use throughout the elective that consisted of surgical instruments, sutures, a Dog Abdominal Surrogate for Instructional Exercise (10), and practice worksheets.

### Course evaluation

Student satisfaction with course content was collected utilizing SurveyMonkey, an online survey instrument, upon completion of each of the 4 weeks. Students were asked to evaluate each session on its relevance, organization, opportunity for discussion, meeting of learning objectives, preparation for residency, and whether the session should remain part of the course in the future, with ratings assigned on a five-point Likert scale.

Data analysis was performed with OpenEpi: Open Source Epidemiologic Statistics for Public Health, Version 2.3.1, and SAS software, Version 9.3 of the SAS System for Windows (SAS Institute Inc., Cary, North Carolina). Statistical analysis was performed using the paired *t*-test for continuous variables and the Kruskal–Wallis one-way analysis of variance and Wilcoxon rank sum test for ranked variables, where appropriate. Values are reported as mean±standard deviation. A *p* value below 0.05 was considered significant.

## Results

### Student assessment

#### Knowledge

There was significant improvement in overall knowledge, as well as improvements in office, gynecology, and obstetrical topics at the completion of the course. Pre- and postcourse scores for the knowledge assessment are demonstrated in Table 2. Student performance on the assessment and management of the eight paging scenarios are demonstrated in Table 3. Students performed well in the assessment and management of acute situations such as an acute abdomen in a postoperative patient. One of the scenarios however was a simple request for Simethicone in a postoperative patient, and the students had the lowest scores for assessment and management for this common case. Paging confidence trended toward improvement for all of the scenarios covered by the paging curriculum.

**Table 2:** Fourth-year medical students’ scores on the pre-and postcourse assessments for the Advanced Clinical Skills in Obstetrics and Gynecology course, University of Michigan, 2013

	Pretest	Posttest	*P*^a^
Knowledge assessment (*N=*5)^b^			
Total	67±5	79±4	<0.01
Office cases	69±7	81±9	0.02
OB	67±6	81±4	<0.01
Gynecology	65±4	75±5	<0.01
Task specific skill assessment (*N=*4)^c^			
Two-handed knot tie without tension	24±7	13±2	0.06
Two-handed surgeon's knot under tension	32±7	18±6	<0.01
Simple interrupted suture	44±8	31±17	0.06
Running simple suture	381±58	248±60	0.06

a Student's paired *t*-test

b data presented as mean scores percent correct±standard deviation

c data presented as mean time in seconds±standard deviation.

**Table 3:** Fourth-year medical students’ scores in the assessment and management of the eight paging scenarios for the Advanced Clinical Skills in Obstetrics and Gynecology course, University of Michigan, 2013 (*N=*5)^a^

	Assessment (%)			Management (%)		
	
Scenario	Mean±SD	Min	Max	Mean±SD	Min	Max
Postpartum vulvar hematoma	80±23	50	100	80±20	60	100
Postcesarean section tachycardia	68±18	45	90	72±22	50	100
Postpartum urinary retention	80±27	50	100	72±33	20	100
Postcesarean IV management	92±13	70	100	76±54	−20	100
Posthysterectomy oliguria	89±25	45	100	70±37	10	100
Posthysterectomy tachycardia	83±13	65	100	40±14	20	60
Postsalpingostomy acute abdomen	100±0	100	100	100±0	100	100
Posthysterectomy gas pain	78±18	50	100	40±55	0	100

a Clinical scenarios and scoring instruments available upon request from the authors. Minimum and maximum scores represent the range of scores received by the five fourth-year medical students.

#### Skills

There was a trend toward improvement in the times for all of the open and laparoscopic skills, with significant improvement demonstrated in the two-handed surgeon's knot under tension and the basic laparoscopy skill on the box trainer. Pre- and postcourse times for the skills assessments are demonstrated in Table 2. Two of the students were not able to complete a one-handed knot at the time of the pretest, and all five students were able to tie a one-handed knot at the time of the posttest.

### Course evaluation

There was a 100% response rate to the online satisfaction survey, and overall satisfaction was very high for all components of the course. The satisfaction scores were highest for the active learning components of the curriculum, specifically the flipped classroom, simulation, anatomic dissections, and operative procedures.

## Discussion

This 4-week elective in Advanced Clinical Skills in Obstetrics and Gynecology led to improvement in knowledge, paging confidence, and surgical skills, and had very high student satisfaction evaluations. These findings, although limited by the small sample in the pilot study, are consistent with findings previously published on general surgery residency preparation electives for fourth-year medical students (11, 12). Many medical schools are currently struggling with the creation of individual curricula in order to meet the Milestones requirement for the various specialties (13), and this pilot experience suggests that a 1-month clinical preparation course can successfully help prepare fourth-year students for the expectations of Milestones One in obstetrics and gynecology.

This program also demonstrates that a comprehensive curriculum can be completed on a relatively low budget, but requires significant faculty time for the active learning components. Our course incorporated a considerable amount of active learning with flipped classroom active learning sessions, simulation, and anatomic dissections. Learner satisfaction data demonstrated higher student satisfaction with these active learning sessions. Although a traditional didactic curriculum may be a seemingly more simple way to meet the minimal standards for compliance with requirements such as Milestones One, active learning leads to better engagement and retention of both knowledge and skills (14). This curriculum presents an example of the use of active learning for preparation for year one of residency.

A limitation of this pilot experience is the small number of students that participated in the course. The Advanced Clinical Skills Course will be offered annually, and we have 13 students enrolled in the course for the upcoming 2014 session. The final ACGME Milestones, released in September 2013, map very similarly to the preliminary APGO/CREOG Milestones on which our curriculum was modeled. The final ACGME Milestones contain more systems-based practice and practice-based learning and improvement components that will be included in our 2014 course. In addition, we are in the process of collaborating with multiple programs to further assess the overall cost and effectiveness of the elective and we plan to survey program directors where students who went through this program match to assess their competency relative to students who did not go through such a course in their fourth year. Future studies will be needed to assess Milestones Level One competencies in learners who participated in a preparation elective compared to those who did not. The organization and implementation of these electives is labor intensive, and future studies will also need to assess whether there are longer term benefits to learners.

In conclusion, this pilot study demonstrates a practical and effective approach for preparing fourth-year medical students for the expectations of Milestones Level One in obstetrics and gynecology. As medical schools and specific specialties work to meet the first step in the ACGME's NAS, this curriculum can serve as a framework for other specialties.

## Previous presentation: poster presentation

CREOG and APGO Annual Meeting, Atlanta, Georgia, February 2014.

## References

[1] ACGME Next Accreditation System http://www.webcitation.org/6RnzAvvKA.

[2] ACGME Milestones for Obstetrics and Gynecology http://www.webcitation.org/6RnzgxXdk.

[3] Barzansky B, Simon F, Brotherton S (2001). The fourth-year medical curriculum: has anything changed in 20 years?. Acad Med.

[4] Walling A, Merando A (2010). The fourth year of medical education: a literature review. Acad Med.

[5] APGO Milestone One Component Map http://www.webcitation.org/6Ro0KNsmN.

[6] LessonLams Community http://www.webcitation.org/6TGB061Rv.

[7] Schwind C, Boehler M, Markwell S, Williams R, Brenner M (2011). Use of simulated pages to prepare medical students for internship and improve patient safety. Acad Med.

[8] Andreatta PB, Frankel J, Smith SB, Marzano D (2011). Interdisciplinary team training identifies discrepancies in institutional policies and practices. Am J Obstet Gyn.

[9] Mashaud LB, Arain NA, Hogg DC, Scott DJ (2013). Development, validation, and implementation of a cost-effective intermediate-level proficiency-based knot-tying and suturing curriculum for surgery residents. J Surg Educ.

[10] DASIE (Dog Abdominal Surrogate for Instructional Exercise) http://www.webcitation.org/6Ro0c2RwX.

[11] Boehler M, Rogers D, Schwind C, Fortune J, Ketchum J, Dunnigton G (2004). A senior elective designed to prepare medical students for surgical residency. Am J Surg.

[12] Tocco N, Brunsvold M, Kabbani L, Lin J, Stansfield B, Mueller D (2013). Innovation in internship preparation: an operative anatomy course increases senior medical students’ knowledge and confidence. Am J Surg.

[13] Santen S, Rademacher N, Heron S, Khandelwal S, Hauff S, Hopson L (2013). How competent are emergency medicine interns for level 1 milestones: who is responsible?. Acad Emerg Med.

[14] Rohrer D, Pashler H (2010). Recent research on human learning challenges conventional instructional strategies. Educ Res.

